# Malignant Gastrointestinal Neuroectodermal Tumor (GNET) Mimicking Small Bowel Lymphoma: A Case Report

**DOI:** 10.7759/cureus.59105

**Published:** 2024-04-26

**Authors:** Yong Jia, Yi Yan, Pamela Hebbard, Gregory Garvin, Miao (Vivian) Lu

**Affiliations:** 1 Department of Pathology, University of Manitoba, Winnipeg, CAN; 2 Department of Radiology, University of Manitoba, Winnipeg, CAN; 3 Department of Medical Imaging, St. Joseph's Health Care, London, CAN; 4 Department of Surgical Oncology, University of Manitoba, Winnipeg, CAN

**Keywords:** bowel lymphoma, mesenchymal neoplasm, ewsr1-atf1, clear cell sarcoma-like tumor of the gastrointestinal tract, malignant gastrointestinal neuroectodermal tumor (gnet)

## Abstract

A malignant gastrointestinal neuroectodermal tumor (GNET) is a rare entity, characterized as a malignant mesenchymal neoplasm occurring exclusively near the gastrointestinal tract, prone to frequent local recurrence and metastasis. Here, we report a case of a 49-year-old male presented with abdominal pain and weight loss. The patient had a remote history of thymic B-cell lymphoma. An abdominal computed tomography (CT) scan revealed a focal wall thickening of the terminal ileum with mesenteric lymphadenopathy, suggestive of lymphoma. A core needle biopsy of the mesenteric node was inconclusive. A right hemicolectomy was subsequently performed. Histologically, abundant multinucleated osteoclast-like giant cells are present. The tumor cells show diffuse strong positivity for S100 and SOX10. EWSR1-ATF1 gene fusion was identified by fluorescence in situ hybridization (FISH), consistent with a diagnosis of GNET. This case emphasizes a diagnostic challenge of a rare malignancy.

## Introduction

A malignant gastrointestinal neuroectodermal tumor (GNET) is a recently described histology entity that was previously referred to as a “clear cell sarcoma-like tumor of the gastrointestinal tract” [1]. It was first identified by Zambrano et al. in 2003 that this tumor shared some features of clear cell sarcoma of the gastrointestinal tract but also had some notable differences, such as the presence of osteoclast-like giant cells and negativity for melanocytic markers [2,3]. More recent studies revealed the specific histologic features as well as evidence of primitive neural crest cell origin of this tumor, suggesting that GNET is a distinct gastrointestinal tract mesenchymal neoplasm [4-6].

It often affects adolescents and young adults with vague clinical presentation and highly aggressive clinical behavior [5,6]. The imaging findings of this entity are extremely nonspecific and under-characterized [6,7]. Morphologically, GNET shows diffuse sheets or nests of large, epithelioid, and polygonal tumor cells with eosinophilic or clear cytoplasm and vesicular chromatin. The presence of multinucleated osteoclast-like giant cells is a distinctive feature. Immunohistochemically, tumor cells almost always show diffuse strong positivity for S100 and SOX10, and negativity for melanocytic markers [2,5,6]. The genetic feature of GNET is described as the EWSR1 rearrangements involving either ATF1 or CREB1 gene [3,8].

Here, we report a case of malignant GNET involving the ileum, which mimics lymphoma clinically and radiologically. The tissue sampling of the excisional biopsy of the mesenteric node was inconclusive. The final diagnosis of GNET was made in the resection specimen.

## Case presentation

A 49-year-old male presented with a five-month history of abdominal pain, profound iron deficiency anemia, weight loss, and fever. The patient also experienced additional symptoms, including fatigue and night sweats. He had a remote history of B-cell lymphoma of the thymus in 2003 in another country and was treated with thymectomy, chemotherapy, and radiation. Given the patient's history of lymphoma, these symptoms were classified as B symptoms commonly associated with lymphoma. Investigations for infectious causes, such as culture and sensitivity tests, were not conducted. Routine biochemistry tests yielded non-contributory results, and the patient's chest X-ray appeared normal.

**Figure 1:**
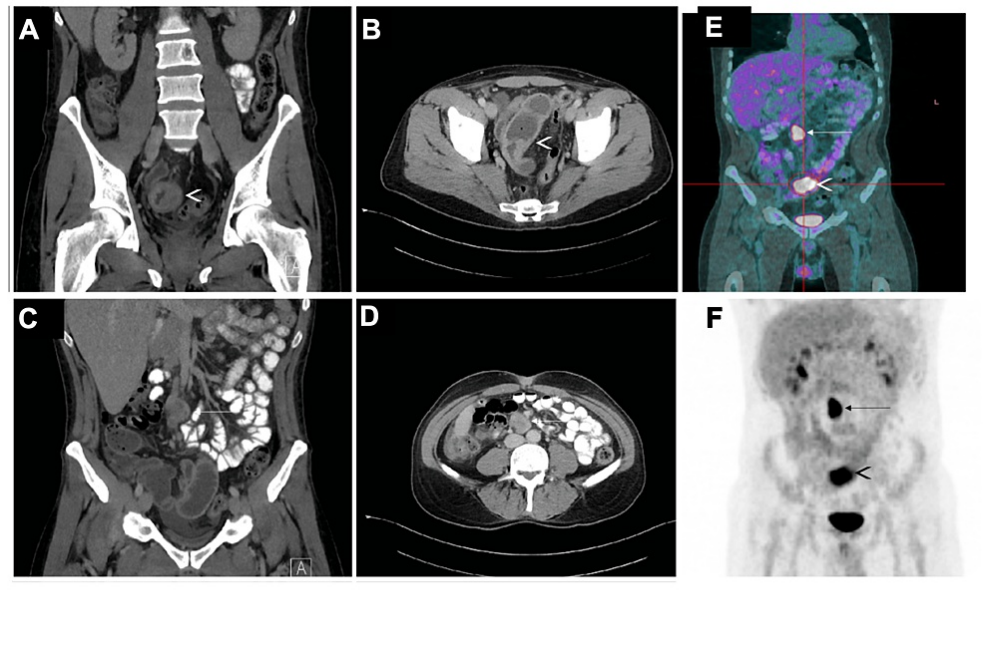
CECT and PET-CT scan of the abdomen. (A) and (B) Coronal and axial images of the CT abdomen showed a small bowel wall thickening (the white arrowhead). The wall measured up to 7 mm in diameter. The small bowel proximal to this level was not dilated. (C) and (D) Coronal and axial images of the CT abdomen showed an enlarged mesmeric lymph node just proximal to the aortic bifurcation (white arrow). (E) and (F) A PET F-18 FDG study was obtained with nondiagnostic CT images for anatomic localization and attenuation correction. Within the abdomen, a mesenteric lymph node at the level of the mid to distal abdominal aorta demonstrated increased metabolic activity within a max SUV of 15.5 (white arrow). The bowel wall measured up to 1.2 cm in thickness and demonstrated increased FDG uptake with a max SUV of 11.3 (arrowhead). CECT, contrast-enhanced computed tomography; FDG, fluorodeoxyglucose; PET-CT, positron emission tomography-computed tomography; SUV, standardized uptake value

Initially, gastrointestinal tract malignancy was suspected as the cause of the patient's anemia. However, a gastroscopy revealed normal findings, and the patient declined colonoscopy. subsequent contrast CT scan of the abdomen demonstrated irregular focal wall thickening of the distal ileum, which was new since the previous CT abdomen/pelvis was performed for diffuse abdominal pain nine months ago (Figures 1A-1B). The bowel wall measured up to 7 mm in thickness, and abnormal thickening spanned 2.5 cm in length. No evidence of upstream bowel obstruction was, however, revealed. In addition, multiple enlarged mesenteric lymph nodes were identified in the right lower quadrant, the largest one just proximal to the aortic bifurcation measuring 2.0 cm x 2.7 cm (Figures 1C-1D). The constellation of findings could represent a manifestation of lymphoma with small bowel involvement or metastatic disease from a primary small bowel tumor. A positron emission tomography (PET) F-18 fluorodeoxyglucose (FDG) scan (PET-CT) was subsequently obtained. Within the abdomen, enlarged lymph nodes were observed along the mid to distal abdominal aorta, exhibiting increased metabolic activity with an SUV of 15.5. Additionally, marked bowel wall thickening involving the distal ileum was noted, demonstrating increased FDG uptake with a maximum SUV of 11.3 (Figures 1E-1F).

A diagnostic laparoscopy and incisional core biopsy of the mesenteric lymph node was initially performed. The specimen showed a small number of intermediate to large atypical cells with vesicular chromatin, prominent nucleoli, and ample cytoplasm, indicating high-grade malignant neoplasm. The neoplastic cells were positive for BCL-2, BCL-6, BCL1, and SOX10 and negative for HMB45, MART-1, and CD117. Unfortunately, definitive tumor typing failed as there was insufficient material for further study.

**Figure 2:**
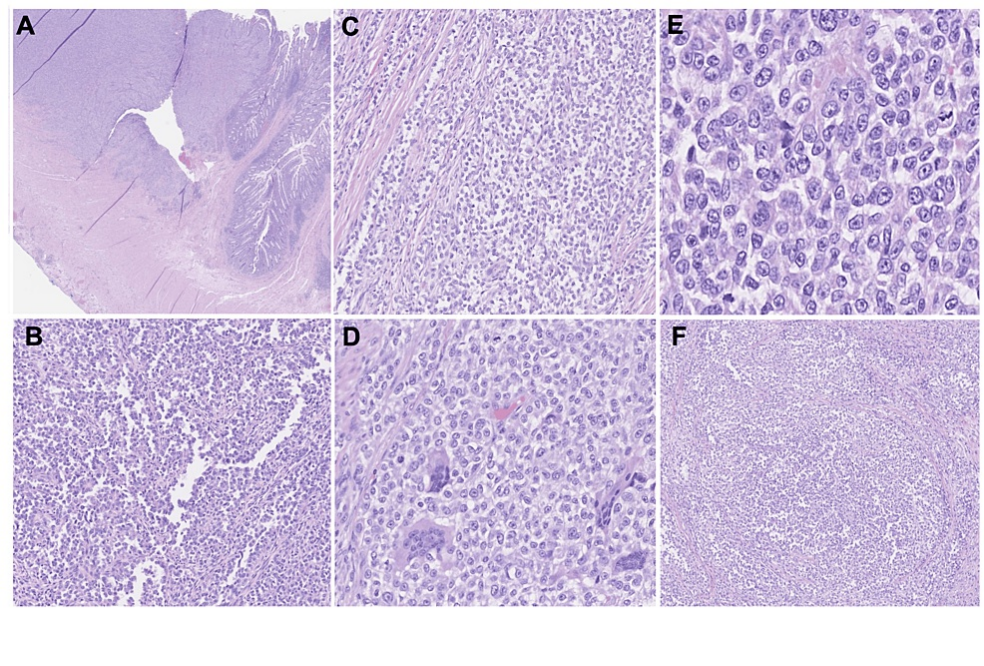
Microscopic findings of malignant gastrointestinal neuroectodermal tumor (GNET). (A) x2 H&E: An ulcerated tumor was seen in the ileum. (B) x4 H&E and (C) x4 H&E: the tumor grows in diffuse sheets and nests with focal pseudopapillary and pseudoalveolar patterns. (D) x10 H&E: Abundant multinucleated osteoclast-like giant cells were present. (E) x20 H&E: The tumor cells had large, epithelioid, and polygonal cells with eosinophilic or clear cytoplasm, vesicular chromatin, and prominent nucleoli. (F) x4 H&E: One of the lymph nodes was partially replaced by the tumor. H&E, hematoxylin and eosin

The patient underwent a right hemicolectomy, revealing a 3.5 cm circumferential and ulcerated tumor involving the full thickness of the ileum (Figure 2A). Multiple tumor-replaced lymph nodes were identified, measuring up to 6 cm in the greatest dimension. The tumor displayed growth in diffuse sheets and nests with focal pseudoalveolar and pseudopapillary patterns (Figures 2B-2C). Tumor necrosis was also observed. The tumor cells exhibited similar cytologic features to those observed in the needle biopsy. They consisted of large, epithelioid, and polygonal cells with eosinophilic or clear cytoplasm, vesicular chromatin, and prominent nucleoli. Additionally, abundant multinucleated osteoclast-like giant cells were present (Figures 2D-2E). The tumor involved five out of 21 regional lymph nodes (Figure 2F).

**Figure 3:**
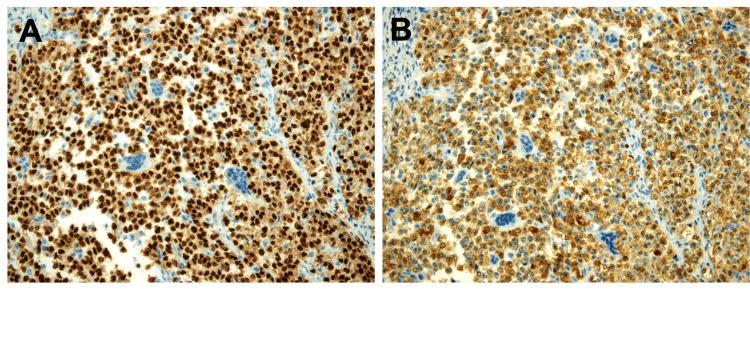
IHC staining of S100 and SOX10. Tumor cells were diffusely and strongly positive for (A, X20) S100 and (B, X20) SOX10. IHC, immunohistochemistry

**Figure 4:**
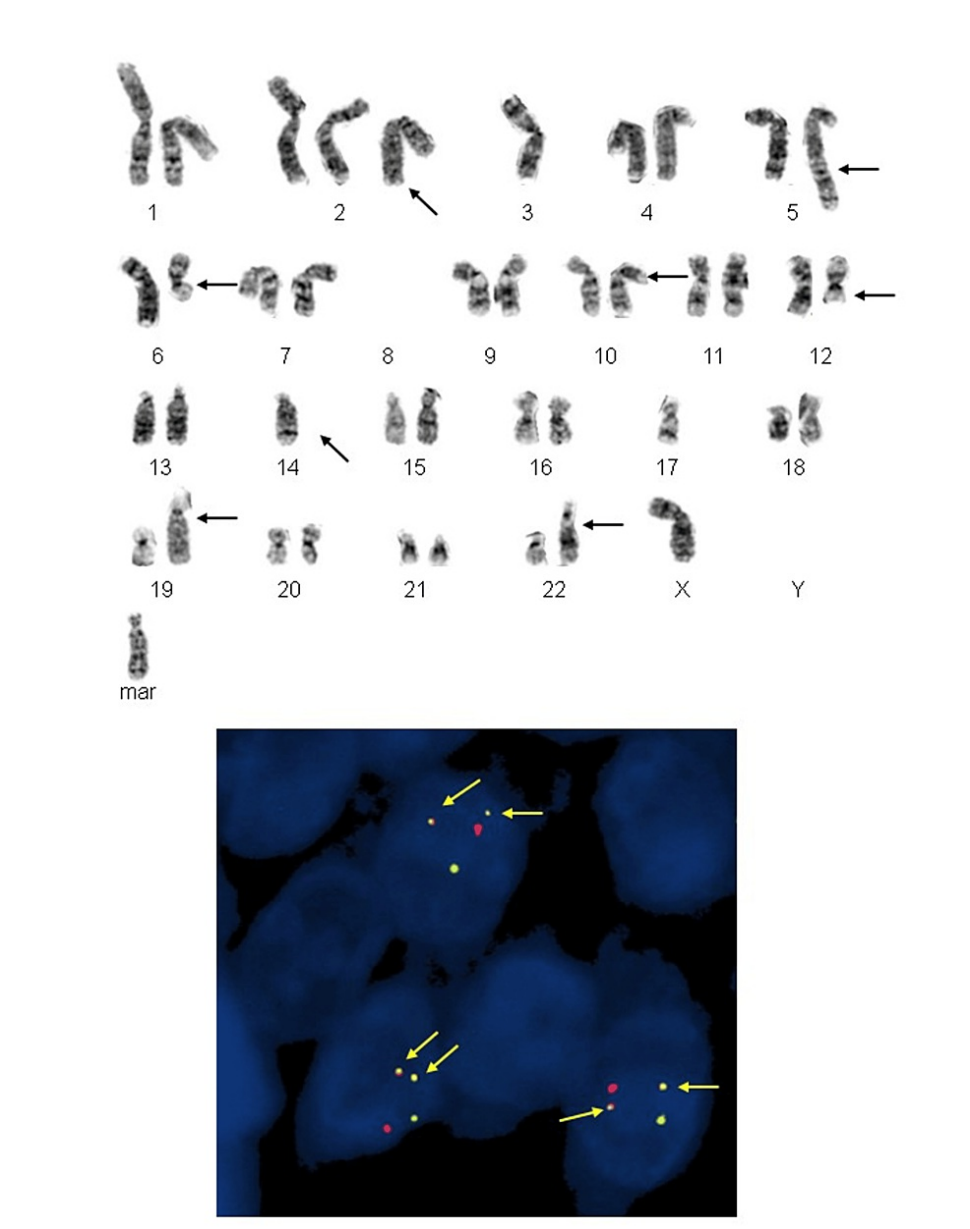
12;22 translocation and EWSR1/ATF1 fusion. Upper: G-banded karyotype illustrating the 12;22 translocation. Note that the marker chromosome in this figure was selected from another metaphase cell preparation from this patient’s tumor for illustration purposes. Lower: The dual-color, dual-fusion EWSR1/ATF1 probe set demonstrated juxtaposed orange-green signals,  indicative of the translocation event (arrows) in neoplastic interphase nuclei.

Tumor cells showed diffuse strong positive for S100 and SOX10 (Figures 3A-3B). The melanocytic differentiation was absent (negative for HMB45, Melan A, and microphthalmia transcription factor). They were also negative for synaptophysin, chromogranin, CD117, DOG1, pancytokeratin, Desmine, and MyoD1. The cytogenetic study showed 47, xy and t(12;22). The 12;22 translocation was associated with EWSR1/ATF1 fusion. The fluorescence in situ hybridization (FISH) assessment was positive for the fusion of the EWSR1 (22q12) and ATF1 (12q13.13) loci in 96.5% of the 200 interphase cells analyzed from the tissue section (Figure 4).

**Figure 5:**
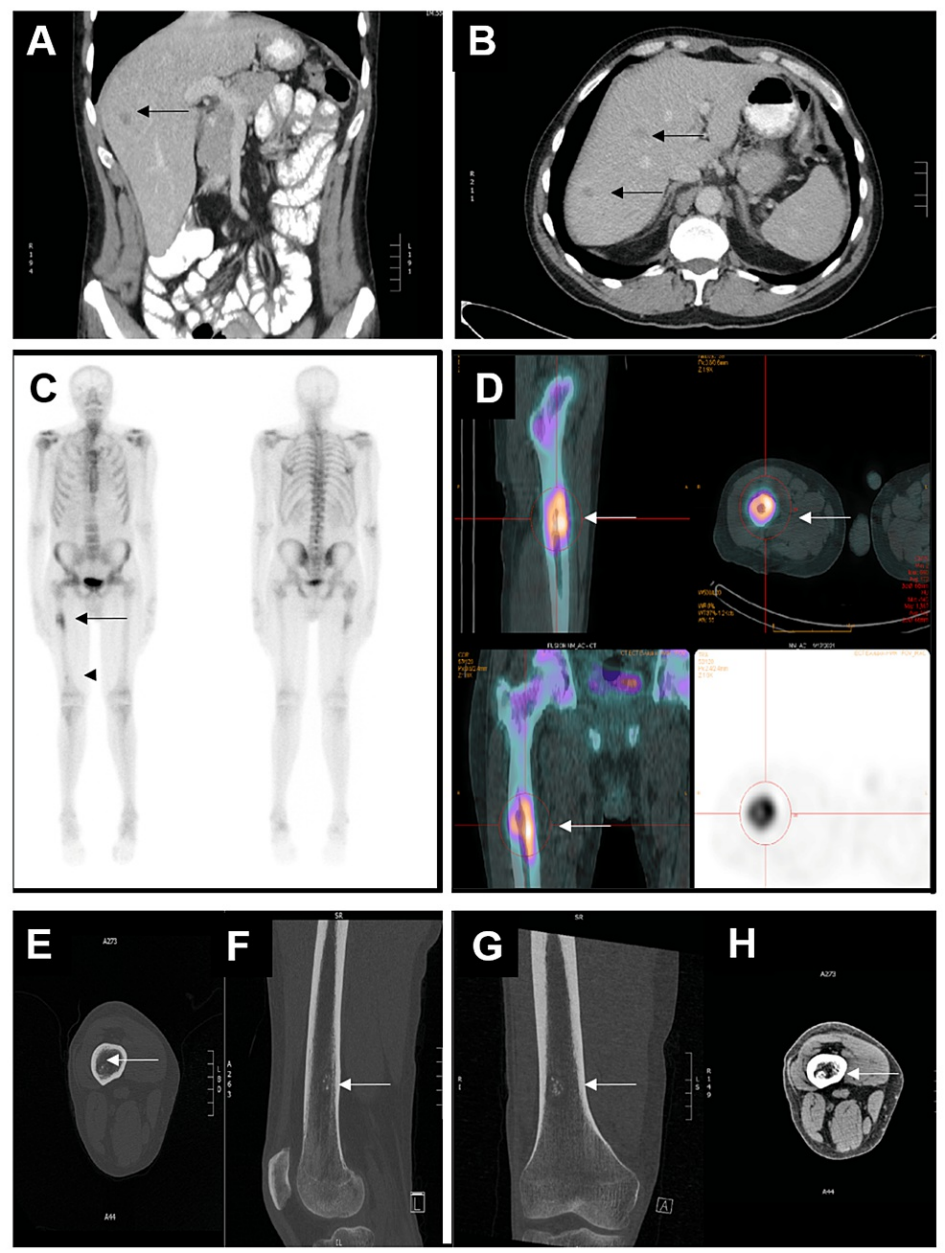
Postoperative follow-up imaging. (A) and (B) A three-month postoperative follow-up CT scan with coronal and axial images showing multiple small hypoattenuating lesions, which were new since previous CT scans, and worrisome for liver metastases. (C) and (D) Follow-up bone scan showed a new intense activity to the proximal third of the right femoral diaphysis corresponding to the lytic lesion with cortical erosion anteriorly and suspected periosteal reaction (black arrow). There was mild focal uptake in the right distal femur (black arrowhead). These were consistent with metastatic disease. (E)-(H) Axial, sagittal, and coronal images of CT scan of lower extremity with bone window (E-G) and soft tissue window (H). There was an area of lucency and sclerosis involving the distal femoral shaft, which correspond to the bone scan abnormality (white arrow). No fractures or soft tissue masses were identified.

The patient received postoperative chemotherapy. In addition, the patient also received a blood transfusion every month due to anemia. Unfortunately, a six-month follow-up CT abdomen scan demonstrated new liver metastatic deposits (Figures 5A-5B). He had also been experiencing pain in his right thigh post-surgery. A bone scan revealed significant uptake in the proximal femur and mild focal uptake in the right distal femur (Figures 5C-5D). A CT scan showed a corresponding lytic lesion with cortical erosion anteriorly and a suspected periosteal reaction in the proximal right femoral diaphysis. Additionally, the distal femoral shaft exhibited an area of lucency and sclerosis (Figures 5E-5H). The findings were consistent with multifocal osseous metastasis. No fractures or soft tissue masses were, however, identified. Orthopedic oncology was, therefore, involved. Given the fact that significant erosion into the anterior femur increases the risk of a fracture, a prophylactic nailing of his femur was performed.

**Figure 6:**
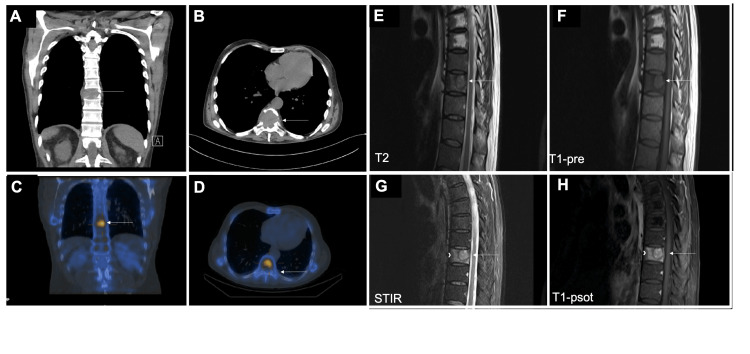
Follow-up imaging. (A) and (B) Post-op follow-up CT chest showed a destructive lytic lesion of the T9 vertebral body with vertebral height loss. There was an associated soft tissue mass (white arrow). (C) and (D) increased activity to T9 corresponding to the large lytic lesion on the CT chest (white arrow). (E)-(H). MRI spine with T2, T1-pre, STIR, and T1-post images showed a T1 hypointense and T2 hyperintense lesion (white arrow) with a heterogeneous enhancement and marked edema (arrowhead), consistent with a metastatic deposit. No obvious cord compression was observed.

Furthermore, a 12-month follow-up chest CT scan showed a destructive lytic lesion of the T9 vertebral body with mild vertebral height loss and an associated soft tissue mass (Figure 6). A corresponding increased activity on bone scan was noted (Figure 6). There was an associated soft tissue mass (Figure 6). The MRI spine confirmed the presence of a T1 hypointense and T2 hyperintense lesion exhibiting heterogeneous enhancement and marked edema, consistent with a metastatic deposit (Figure 6). No obvious cord compression was identified. Due to the widely metastatic liver and osseous diseases, he was re-categorized as stage 4 metastatic disease now and received palliative radiation and chemotherapy. Unfortunately, he passed away 15 months post-surgery.

## Discussion

GNET is an extremely rare sarcoma, with only 90 cases published in the English literature up to 2020 [1,6]. It occurs predominantly in adolescents and younger adults with a median age of 36 years [6]. GNET exclusively occurs in or near the gastrointestinal tract. The reported tumor locations range from the oral cavity to the colon and peritoneum, but the small intestine, especially the ileum, remains the most commonly affected site [6,9]. It is highly aggressive with a high risk of regional lymph node or liver metastasis and recurrence [1,5,10,11]. The etiology of GNET remains unknown. However, there are three reported cases with a remote history of childhood malignancy (hepatoblastoma, Ewing sarcoma, and neuroblastoma, respectively), along with prior chemotherapy and radiation treatment [12-14]. Our patient had a remote history of lymphoma and received chemotherapy and radiation, which raises the possibility of chemoradiation as a contributing factor of GNET in our case.

Very few studies describe the imaging features of GNET. It could present either as an intraluminal mass lesion arising from a bowel wall [6,15] or a focal bowel wall thickening on the CT scan, which is nonspecific [16,17]. No MRI features are investigated. In our case, a new small bowel wall thickening was favored to represent early lymphoma involvement given the remote history. A new primary malignancy, localized inflammatory disease, or infection are also possible differential diagnoses.

Grossly, most GNET growths present as transmural mass with mucosal ulceration and subserosa involvement. The medium tumor size is 4.5 cm, ranging from 1.5 to 13.5 cm, with usually a uniform fleshy cut surface. Due to its rarity and overlapping features with a variety of GI neoplasms, GNET can be misdiagnosed. The diagnosis might become even more challenging in limited tissue specimens, like the needle core biopsy of our reported case. Histologically, GNET shared features of conventional clear cell sarcoma of the gastrointestinal tract (CCS-GI). However, one characteristic feature of GNET is the presence of CD68-positive multinucleated osteoclast-like giant cells [18-20]. Due to the neuroectodermal differentiation, GNET usually reveals strong expression of S100 and SOX10 as well as other neuroendocrine markers, such as synaptophysin (56%), NB84 (50%), and NSE (45%) [1,11,21]. Other differential diagnoses may include malignant peripheral nerve sheath tumor (MPNST), metastatic melanoma, alveolar rhabdomyosarcoma, metastatic clear cell carcinoma, lymphoma, and gastric granular cell tumor [5,21-25]. The absence of melanin pigment in GNET might point away from conventional CCS-GI and melanoma. The diagnosis might become even more challenging when receiving an FNA sample. Rarely, under such circumstances, GNET might be misinterpreted as a benign tumor. Boland and Folpe reported a case of stomach GNET with oncocytic cytoplasmic change that was initially diagnosed with a benign granular cell tumor on an FNA specimen [22].

*EWSR1* gene has been identified as a partner gene in a wide variety of sarcomas. The majority of GNETs (81%) contain EWSR1 rearrangements, such as EWSR1-ATF1 or EWSR1-CREB1 [3,8,11,26]. Molecular studies are particularly helpful in certain challenging cases. For example, alveolar rhabdomyosarcoma often demonstrates t(2;13) or t(1;13) and MPNST usually lacks EWSR1-ATF1/CREB1 fusions. Interestingly, conventional CCS-GI shares the same genetic alterations raising the debate on the close origin between conventional CCS-GI and GNET [2,23]. Regardless, the diagnosis of GNET should not rely solely on molecular assessment.

## Conclusions

In summary, we have reported a case of a malignant GNET arising from the ileum. Raising awareness of this extremely rare but histologically and molecularly distinctive sarcoma is important due to its highly aggressive behavior and poor clinical outcome. Indeed, our case had developed liver and osseous metastases in short follow-up studies. While correct imaging and pathological diagnosis could be challenging, it is essential to understand this disease and develop potential targeted therapy.
